# Machine learning for chemotherapy decision-making in breast cancer using large language model

**DOI:** 10.3389/fdgth.2026.1738346

**Published:** 2026-07-06

**Authors:** Md Serajun Nabi, Dema Yuden, Thinley Yeshey Choden, S. M. Asiful Islam Saky, Hasanul Bannah, Md Sabbir Hossen, Mohammad Faizal Ahmad Fauzi, Hezerul Bin Abdul Karim

**Affiliations:** 1 Faculty of Artificial Intelligence and Engineering, Multimedia University, Cyberjaya, Malaysia; 2 School of Computing and Informatics, Albukhary International University, Alor Setar, Kedah, Malaysia; 3 School of Digital Health, KPJU Healthcare University, Nilai, Negeri Sembilan, Malaysia

**Keywords:** digital health, breast cancer, AI for medicine, chemotherapy decision support, causal inference, causal survival analysis, individualized treatment effect estimation, precision oncology

## Abstract

**Introduction:**

Breast cancer chemotherapy decision-making remains challenging due to biological heterogeneity and variability in clinical practice. This study proposes a hybrid framework integrating machine learning (ML), causal reasoning, and large language models (LLMs) to improve treatment recommendations.

**Methods:**

Using the METABRIC dataset, eleven pre-treatment clinicopathologic variables were selected. A Random Forest classifier was developed and compared with baseline ML models. Individualized treatment benefit was estimated through inverse probability-weighted causal survival analysis, while GPT-4 was employed using few-shot prompting to generate clinical rationales.

**Results:**

The Random Forest achieved an AUC of 0.91, outperforming benchmark models. Causal analysis identified heterogeneous treatment benefits and patient groups where chemotherapy could potentially be deprioritized. GPT-4 showed moderate agreement with the Random Forest (Cohen's κ = 0.13) while consistently highlighting clinically relevant factors. Uplift-based ML policies outperformed treat-all and treat-none strategies, and GPT-4 improved interpretability through rationale-driven explanations.

**Discussion:**

By combining predictive ML, causal survival modeling, and LLM-based rationale generation, the proposed framework provides a promising approach for personalized and transparent chemotherapy decision support in oncology.

## 1 Introduction

Breast cancer is one of the most common cancers worldwide and continues to be a leading cause of cancer deaths among women (1, 2). Treatment choices, especially regarding chemotherapy, significantly affect long-term outcomes. Traditionally, eligibility for chemotherapy is based on specific clinical and pathological factors, such as tumor size, histologic grade, lymph node involvement, and biomarker status, including estrogen receptor (ER), progesterone receptor (PR), and human epidermal growth factor receptor 2 (HER2) (3, 4). Although hormone receptor and HER2 status are included in treatment guidelines, there is still a wide range of variability in how treatments are assigned (5, 6). This can lead to some patients receiving unnecessary treatment while others do not get adequate care (7). This uncertainty highlights the need for better tools to personalize chemotherapy recommendations. Standard predictive methods, like regression-based nomograms and newer machine learning (ML) classifiers, have mainly focused on predicting recurrence or survival rates (8, 9). While these models can categorize patients into risk groups, they often do not adequately assess the specific benefits of chemotherapy for individuals. Additionally, many ML models operate as “black boxes,” which makes them hard to interpret. This lack of clarity can hinder their use in clinical settings where trust and accountability are crucial (10).

Recent advances in large language models (LLMs) have introduced new opportunities for enhancing clinical decision support by generating human-readable explanations and synthesizing complex medical knowledge. However, the suggested potential utility of LLMs depends heavily on the prompting strategy used to guide their outputs. Zero-shot prompting, where the model receives only a task description without examples, enables rapid deployment but often lacks domain-specific grounding (11, 12). Few-shot prompting, which provides a small number of task-relevant examples, improves consistency and aligns outputs with expected clinical reasoning patterns, and is the approach adopted in this study (11, 13). Chain-of-thought (CoT) prompting further enhances interpretability by encouraging step-by-step reasoning, which has shown promise in complex medical decision-making tasks (14). More advanced variants, including self-consistency CoT, tree-of-thought prompting, and retrieval-augmented generation (RAG), aim to improve reasoning robustness and factual grounding, though they introduce additional computational and implementation complexity (15). Importantly, in high-stakes clinical settings, structured prompting approaches are often preferred due to their improved reproducibility and reduced variance in outputs.

Beyond prompting strategies, LLMs are increasingly being explored across a wide range of oncology applications. These include tumour board decision support, where models have demonstrated partial concordance with multidisciplinary expert recommendations (16, 17); clinical trial matching through automated parsing of eligibility criteria (18); radiology and pathology report summarization; and precision oncology tasks such as synthesizing genomic and clinical evidence for treatment planning (19, 20). These systems offer the potential to augment clinical expertise, particularly in settings where access to subspecialty knowledge is limited. However, most existing studies remain descriptive or concordance-based and do not evaluate downstream clinical impact, highlighting the need for frameworks that connect LLM outputs with measurable patient-level outcomes.

Traditional decision support techniques, like regression-based predictive models and more recent machine learning (ML) classifiers, have primarily sought to predict outcomes with little interest in estimating the individualized value of chemotherapy (8, 9). Furthermore, most ML methods are black-box models that limit their application in clinical settings where explanation and interpretability are paramount to gaining acceptance and trust (10). This points to a core deficit, decision-support models that combine predictive capability with patient-level reasoning aligned with oncology practice.

While prior work has explored machine learning prediction, causal inference for treatment effect estimation, and LLM-based reasoning largely in isolation, there remains a lack of integrated frameworks that connect these components into a unified decision-support pipeline. In oncology specifically, most existing studies evaluate LLM performance using concordance with expert recommendations rather than assessing whether such outputs translate into improved patient-level outcomes. In this study, we propose a hybrid decision-support framework that integrates machine learning prediction, causal survival modeling, and large language models (LLMs) for interpretable chemotherapy recommendation analysis. A Random Forest classifier is trained using pre-treatment clinical and pathological variables to simulate chemotherapy decisions, followed by causal survival uplift modeling with inverse probability weighting (IPW) to estimate individualized treatment benefit over a five-year horizon. GPT-4 is then incorporated as a rationale-generating module to produce structured, clinically aligned explanations that complement the quantitative outputs.

## 2 Literature review

Treatment options for breast cancer increasingly involve molecular testing alongside clinical-pathologic characteristics. The 70-gene MammaPrint test, validated in the MINDACT trial, demonstrated that nearly half of clinically high-risk patients would be downstaged to low risk and can avoid chemotherapy with safety without compromising survival rates (21, 22). It is similarly so with the 21-gene Oncotype DX recurrence score that has been widely employed to rank adjuvant chemotherapy options, especially in ER-positive disease (23). While these assays have good prognostic and predictive value, their cost and limited availability restrict their widespread application in routine care (24).

Consequently, machine learning (ML) techniques have been utilized to imitate or complement genomic tests using standard clinicopathological data. Random forest, gradient boosting, and neural network analyses demonstrated better performance compared with standard regression models for treatment and survival predictions (25, 26). But such models are typically predictive, returning probabilities without a measure of the causal treatment effect. More recent causal inference developments, such as causal forests, targeted maximum likelihood estimation, and uplift modeling, bridge this gap by directly estimating heterogeneous treatment effects (27, 28). Wager and Athey (29), for example, developed causal forests in order to provide patient-specific treatment benefits, while (30) demonstrated their ability in real-world heterogeneous intervention effects.

Table 1 summarizes recent and foundational studies on breast cancer risk stratification, chemo benefit, heterogeneous treatment effect (HTE) estimation, causal uplift, and large language models in clinical decision support. For each study, we highlight the task or setting, main approach, and specific limitations or technical challenges that relate to our pipeline.

**Table 1:** Summary of recent studies on breast cancer prognosis, causal effect estimation, and LLM-based decision support.

Study	Dataset	Method	Task	Key limitations and challenges
Curtis et al. (31)	METABRIC	Genomic clustering, expression profiling	Subtyping, risk stratification	Observational; not treatment-effect focused; no causal inference.
Parker et al. (32)	PAM50 signatures	Supervised risk predictor (PAM50)	Prognosis, therapy guidance	Individualized chemo benefit; no survival benefit estimation.
Sparano et al. (23)	TAILORx	21-gene recurrence score	Prospective chemo de-escalation	Trial-limited; only HR+/HER2–; not ML generalizable.
Wager and Athey (29)	Simulated + applied datasets	Causal Forests	CATE estimation (HTE)	Survival endpoints not directly modeled; needs overlap assumption.
Künzel et al. (33)	Benchmark HTE	T-/X-/S- Learners (meta-learners)	HTE/CATE estimation	Sensitive to base learner bias; calibration required.
Austin (34)	Propensity score matched samples	SMD diagnostics	Covariate balance checking	Diagnostic only; cannot prove unconfoundedness.
Prentice and Zhao (35)	Clinical trial survival data	Cox proportional hazards	Survival modeling (IPTW)	PH assumption; sensitive to censoring/ties; needs robust weighting.
Li et al. (36)	Clinicopathologic	ML classifiers	Predicting pCR to neoadjuvant chemo	pCR surrogate outcome; not uplift; limited external validity.
Nori et al. (13)	Benchmark medical exams	GPT-4 zero/few-shot	Medical knowledge reasoning	Not treatment-grounded; hallucination risks.
Singhal et al. (18)	Med-PaLM dataset	Instruction-tuned LLM	QA and guideline reasoning	Still behind clinicians; safety guardrails needed.
Benary et al. (17)	Synthetic oncology vignettes	GPT-3/4 prompting	Treatment suggestions	Synthetic inputs; limited real-world alignment.
Nabieva et al. (37)	Breast cancer expert cases	ChatGPT	Agreement with experts	Low agreement in HER2/BRCA subgroups; rationale inconsistency.
Stalp et al. (38)	Breast cancer therapy tasks	ChatGPT outputs	Quality of recommendations	Edge-case inconsistency.
Salditt et al. (39)	Review/tutorial	Meta-learners for HTE	Methodological guidance	Emphasizes diagnostics; shows sensitivity to data shift.

In parallel, large language models (LLMs) such as GPT-3.5 and GPT-4 are beginning to transform clinical decision support. Initial trials have shown strong concordance with guideline-recommended oncology treatment advice and even tumor board decisions (16). Further research highlights GPT-4’s ability to provide explanations of reasoning, pushing transparency beyond “black box” ML predictions (19). Few-shot prompting and explanation extraction have been explored in other research, demonstrating that LLMs are able to explain their recommendations in clinically relevant language (20, 40). These efforts have largely been pursued in isolation from the others, with minimal systematic interaction with survival modeling or treatment effect estimation for individuals.

The Table 1 highlights three consistent gaps. Most ML studies focus on optimizing prediction instead of individualized treatment effects. Causal-effect papers seldom connect to survival endpoints in oncology cohorts. LLM evaluations often lack structured prompting that follows guidelines and includes quantitative policy value. Our pipeline directly addresses these gaps by combining a calibrated RF baseline with IPTW-Cox uplift and a few-shot GPT-4 policy. This approach generates auditable rationales. It allows for head-to-head policy evaluation rather than just descriptive concordance. Overall, this positions our results as complementary to previous evidence while tackling known methodological and translation challenges.

While prior work has explored ML prediction and LLM reasoning separately, systematic integration with causal survival analysis for individualized chemotherapy guidance remains underexplored. Stacking Random Forest predictions with causal inference and cross-validating rationale alignment by GPT-4 against biological markers such as ER, PR, and HER2, our study offers a hybrid model that provides quantitative accuracy as well as interpretability. This hybrid approach addresses the twofold oncology decision support challenge, providing individualized benefit estimation while ensuring that recommendations are clinically transparent and reliable.

## 3 Method

### 3.1 Data acquisition and preparation

**Figure 1:**
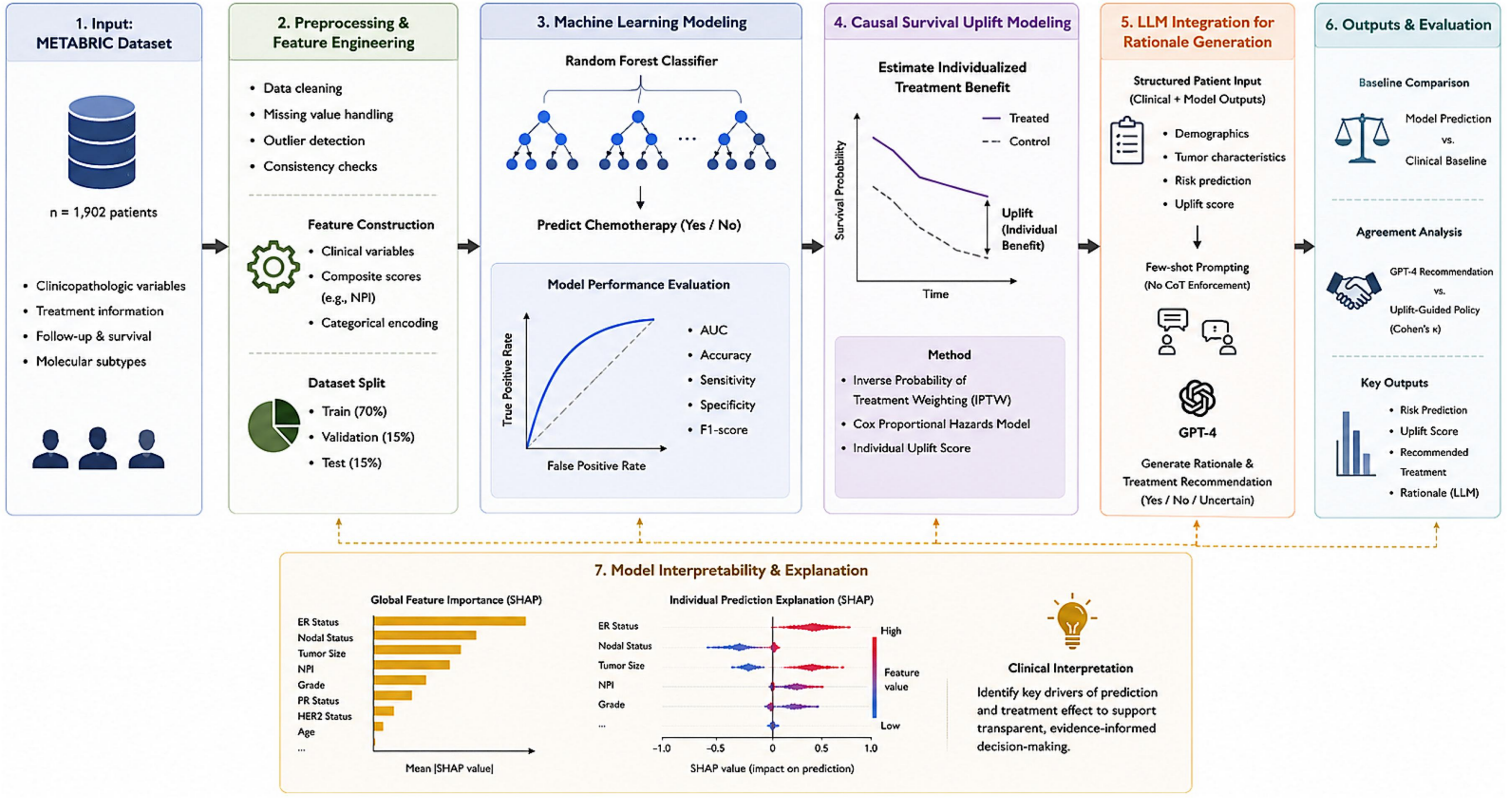
Overview of the proposed framework for breast cancer chemotherapy decision support. The METABRIC dataset was used for preprocessing, feature engineering, Random Forest-based chemotherapy prediction, and causal survival uplift estimation. GPT-4 was integrated using few-shot prompting to generate treatment rationales, while SHAP analysis was applied to support model interpretability.

We used the METABRIC cohort dataset from Kaggle (41), which includes 2,509 patients and 34 variables. We limited the predictors to information available before treatment decisions. These included age, inferred menopausal state, tumor size and stage, positive nodes examined, histologic grade, Nottingham Prognostic Index, ER/PR/HER2 by IHC, and HER2 status by SNP6. We harmonized labels, such as changing “Positive” to “Positive.” We standardized categorical vocabularies and created a signal specific to the cohort for assay discordance (HER2 IHC negative with SNP6 gain). To keep the sample size while recognizing uncertainty, we set categorical missing values, including the block of around 529 cases covering PR/HER2/subtype/therapy fields, to “Unknown.” We median-imputed numerical gaps and standardized continuous features as necessary. We defined survival targets as overall survival in months and an event indicator (deceased = 1). This retained 1,981 patients with complete survival information for time-to-event analyses. We excluded all outcome columns, such as survival, vital status, and therapies, from the feature matrix to prevent leakage. We encoded biomarkers and treatments into numeric form, using binary or one-hot encoding as suitable. We split the data with fixed seeds into stratified train, validation, and test sets (70/15/15) based on chemotherapy label for classification and by treatment assignment for uplift modeling. This ensured a balance of pre-treatment covariates. The overall workflow of our study is summarized in Figure 1.

### 3.2 Data acquisition and preparation

We used the METABRIC cohort dataset from Kaggle (41), which includes 2,509 patients and 34 variables. We limited the predictors to information available before treatment decisions. These included age, inferred menopausal state, tumor size and stage, positive nodes examined, histologic grade, Nottingham Prognostic Index, ER/PR/HER2 by IHC, and HER2 status by SNP6. We harmonized labels, such as changing “Positive” to “Positive.” We standardized categorical vocabularies and created a signal specific to the cohort for assay discordance (HER2 IHC negative with SNP6 gain). To retain sample size while accounting for incomplete records, categorical missing values (including approximately 529 patients with missing PR/HER2/subtype/therapy fields) were encoded as a separate “Unknown” category rather than excluded or imputed. This approach avoids potential selection bias from case-wise deletion but implicitly treats missingness as an informative category, which may introduce bias if the missingness mechanism deviates from the missing-at-random assumption. We median-imputed numerical gaps and standardized continuous features as necessary. We defined survival targets as overall survival in months and an event indicator (deceased = 1). This retained 1,981 patients with complete survival information for time-to-event analyses. We excluded all outcome columns, such as survival, vital status, and therapies, from the feature matrix to prevent leakage. We encoded biomarkers and treatments into numeric form, using binary or one-hot encoding as suitable. We split the data with fixed seeds into stratified train, validation, and test sets (70/15/15) based on chemotherapy label for classification and by treatment assignment for uplift modeling. This ensured a balance of pre-treatment covariates. The overall workflow of our study is summarized in Figure 1.

The dataset exhibited class imbalance (approximately 20% chemotherapy vs. 80% no-chemotherapy), which was addressed through a multi-pronged strategy. First, inverse class weighting was applied within the Random Forest to up-weight the minority chemotherapy class during training. Second, cost-sensitive threshold selection was performed on the validation set using the $F_{\beta }$ metric (with β=2) to prioritise recall, reflecting the higher clinical cost of false negatives (missed chemotherapy candidates) relative to false positives. Third, stratified splitting was employed to preserve class proportions across training, validation, and test sets. In addition to ROC-AUC, the area under the Precision–Recall Curve (AUPRC) was computed to provide a more informative evaluation under class imbalance.

### 3.3 Random forest for chemotherapy decision modeling

To simulate clinical decision-making for chemotherapy administration. We frame “chemotherapy indicated” as a binary choice based on various pre-treatment factors like age, tumor burden, grade, nodal status, NPI, and ER/PR/HER2, including SNP6. Given the heterogeneity in biological features and potential nonlinear interactions among variables, a random forest model was selected due to its robustness to feature types, ability to capture complex patterns, and interpretability via feature importance metrics, which is also effective with different types of data and needs minimal preparation (42). Random Forests provide stable out-of-bag error estimates, need little adjustment, and allow for detailed insight into features without assuming a simple linear relationship (43). This is a clear benefit over basic generalized linear models for this group.

#### 3.3.1 Learning objective and decision rule

Using impurity-reducing splits, each tree divides the covariate space until the terminal leaves have almost homogeneous labels. The empirical positive rate in each leaf is known as the class posterior. The average of the ensemble is the forest probability, Equation 1:

$$\hat{\;p}(x)=\frac{1}{T}\sum_{t=1}^{T}{\hat{\;p}}^{t}(x)$$ (1)

and we convert scores to recommendations with a cost-sensitive threshold $\hat{y}(x;\tau )=1\{\hat{\;p}(x)\geq \tau \}$. To encourage the correct identification of patients who receive chemotherapy, we choose τ on the validation set by maximizing a recall-weighted utility’ Equation 2:

$$\tau^{⋆}=\arg\max_{\tau }F_{\beta }(\tau ),\;\beta >1$$ (2)

and fixed for test evaluation. In our runs, this yields τ≈0.40. Within nodes, we use the standard Gini criterion, Equation 3:

$$G(S)=\sum_{k\in \{0,1\}}p_{k}(1-p_{k}),$$ (3)

with class weights that are inverse to prevalence to counter the 20:80 treated to untreated imbalance. Splits maximize the weighted decrease in G. Because tree models do not depend on scale, continuous features enter on their native scale after median imputation. Categorical variables are label-encoded only to create an ordering for splits. All features are strictly pre-treatment to prevent leakage. For a patient x, the forest probability is the average of leaf posteriors, Equation 4:

$$\hat{\;p}(x)=\frac{1}{T}\sum_{t=1}^{T}{\hat{\;p}}^{\left(t\right)}(x),\;\hat{y}(x;\tau )=1\{\hat{\;p}(x)\geq \tau \}.$$ (4)

Increasing T reduces variance of $\hat{\;p}(x)$ and stabilizes operating points.

#### 3.3.2 Model configuration

To accommodate for treatment label skew, scikit-learn with class-weight balancing was used to create the Random Forest. To minimise variance, the number of trees was raised to 300, and the maximum depth was left unconstrained in order to take full advantage of data-driven splits. The main hyperparameters utilised for training are compiled in Table 2.

**Table 2:** Random forest hyperparameter settings.

Hyperparameter	Value	Description
Number of Trees	300	Ensemble size
Criterion	Gini	Split quality metric
Max Depth	None	Unrestricted tree depth
Min Samples per Split	2	Minimum samples to create a split
Class Weight	Balanced	Adjusts for label imbalance
Random Seed	42	Ensures reproducibility

Three elements that are essential to our study are highlighted by the Algorithm 1. First, in accordance with clinical priorities, validation-guided thresholding (Step 3) guarantees sensitivity to patients who are truly receiving chemotherapy. Second, the strong class skew ≈20:80 is addressed by explicit balancing. Lastly, dual importance measures (Step 5) yield both model-agnostic (permutation) and in-model (Gini) rankings.

Algorithm 1 **Random Forest Training with Threshold Calibration**

**Require:** Pre-treatment features *X*, labels y∈{0,1}; predictors: {Age, Menopause, Tumor Size, Tumor Stage, Nodes+, NPI, Histologic Grade, ER_IHC, PR_IHC, HER2_IHC, HER2_SNP6, HER2_discordant}; n_estimatorsT=300; *random_state* = 42

 1: **Preprocess:**

  1.1 Label-encode categorical columns; median-impute continuous.

  1.2 Drop treatment/outcome fields {Chemo, Hormone, Radio, time, event}.

  1.3 Stratified split: 80% train, 20% test; carve 10–20% of train as validation.

 2: **Fit RF:**

  2.1 Instantiate RandomForestClassifier (n_estimators=300, *class_weight* = “balanced”,*random_state* = 42).

  2.2 Train on training split.

 3: **Threshold selection (validation):**

  3.1 Compute probabilities $\hat{\;p}$ on validation; sweep τ∈[0,1].

  3.2 Select τ maximizing $F_{\beta }$ (β>1 to prioritize recall for treated).

   (In our runs, τ≈0.40.) Freeze τ.

 4: **Final evaluation (test):**

  4.1 Predict $\hat{\;p}$, $\hat{y}=1\{\hat{\;p}\geq \tau \}$; report accuracy, precision/recall/F1, ROC–AUC.

  4.2 Compute permutation importance on validation/test for interpretability.

 5: **Outputs:**

  Trained RF *f*_RF_, fixed threshold τ, and feature-importance rankings (Gini and permutation).

**Ensure:** Reproducible model with class-imbalance handling and calibrated τ.

### 3.4 Causal survival uplift

To estimate the benefit of chemotherapy for each patient, we used inverse probability weighting (IPW) within a Cox proportional hazards model. Let T represent treatment assignment (T=1 for chemotherapy, T=0 for other treatments), X denote patient covariates, and Y be the time-to-event outcome. We estimated the propensity score e(X)=P(T=1∣X) using logistic regression. We then weighted the survival models by Equation 5:

$$w_{i}=\left\{ \begin{array}{cc} \frac{1}{e(X_{i})},\; & T_{i}=1\;\\ \frac{1}{1-e(X_{i})},\; & T_{i}=0\; \end{array}\right.$$ (5)

#### 3.4.1 Causal assumptions and limitations

The validity of IPTW survival models depends on several assumptions. First, unconfoundedness assumes that all relevant factors affecting treatment assignment and survival are captured in X. In retrospective datasets like METABRIC, unmeasured factors such as comorbidities, socioeconomic status, or missing genomic features can still bias estimates. Second, the positivity assumption requires overlap in treatment probabilities across covariates. In some subgroups, such as HER2-positive patients, treatment assignment was almost deterministic. This limits the reliability of uplift estimates. Third, IPTW relies on accurately specified propensity models. Small errors in estimation can bias results, especially with skewed treatment ratios. Finally, the Cox model assumes proportional hazards. This may not strictly hold over long-term survival. Extreme weights can also increase the variance in estimates. These issues suggest that our uplift findings should be viewed as exploratory signals instead of definitive causal effects. External or prospective validation is still needed.

#### 3.4.2 Diagnostics and model insights

To show that the model is neither overfit nor underfit, we present a single figure with the out-of-bag error curve and the held-out test error based on the number of trees. When the curves converge and agree, it indicates stable generalization. To explain what the model learns from pre-treatment biology, we compute permutation importances on the validation split. In our cohort, these consistently highlight nodal burden, tumor size, histologic grade, stage, and ER/HER2 status. This matches clinical understanding and sets the stage for the survival-uplift analysis that follows.

### 3.5 LLM-based clinical decision rationale modeling

In this study, GPT-4 was used not as a predictive or decision-making model but as a tool to create natural-language explanations. Given a patient’s details and the results from the machine learning models, GPT-4 was asked to produce brief text that explained which features were most relevant and why a specific treatment effect might occur. The goal was to make the model outputs easier to understand and more transparent, helping clinicians grasp the algorithm’s reasoning without taking away their clinical judgment.

To achieve this, we developed a structured prompting process that mimics human clinical reasoning without giving independent treatment recommendations. The system takes organized patient data and the related machine learning predictions, then requests the LLM to provide narrative justifications that match the predicted effect. We used a few-shot prompting technique to prepare GPT-4 with examples of believable treatment explanations based on patient characteristics, like hormone receptor status, HER2 score, and tumor grade. The mapping can be summarized as Equation 6:

$$\text{LLM}\;:\;(x,\hat{y})\to \text{Rationale}(x,\hat{y})$$ (6)

where x is a vector of pre-treatment features, $\hat{y}$ is the model-predicted treatment effect, and the output is a textual justification supporting interpretability of the machine learning results.

#### 3.5.1 Prompt construction and few-shot framework

**Figure 2:**
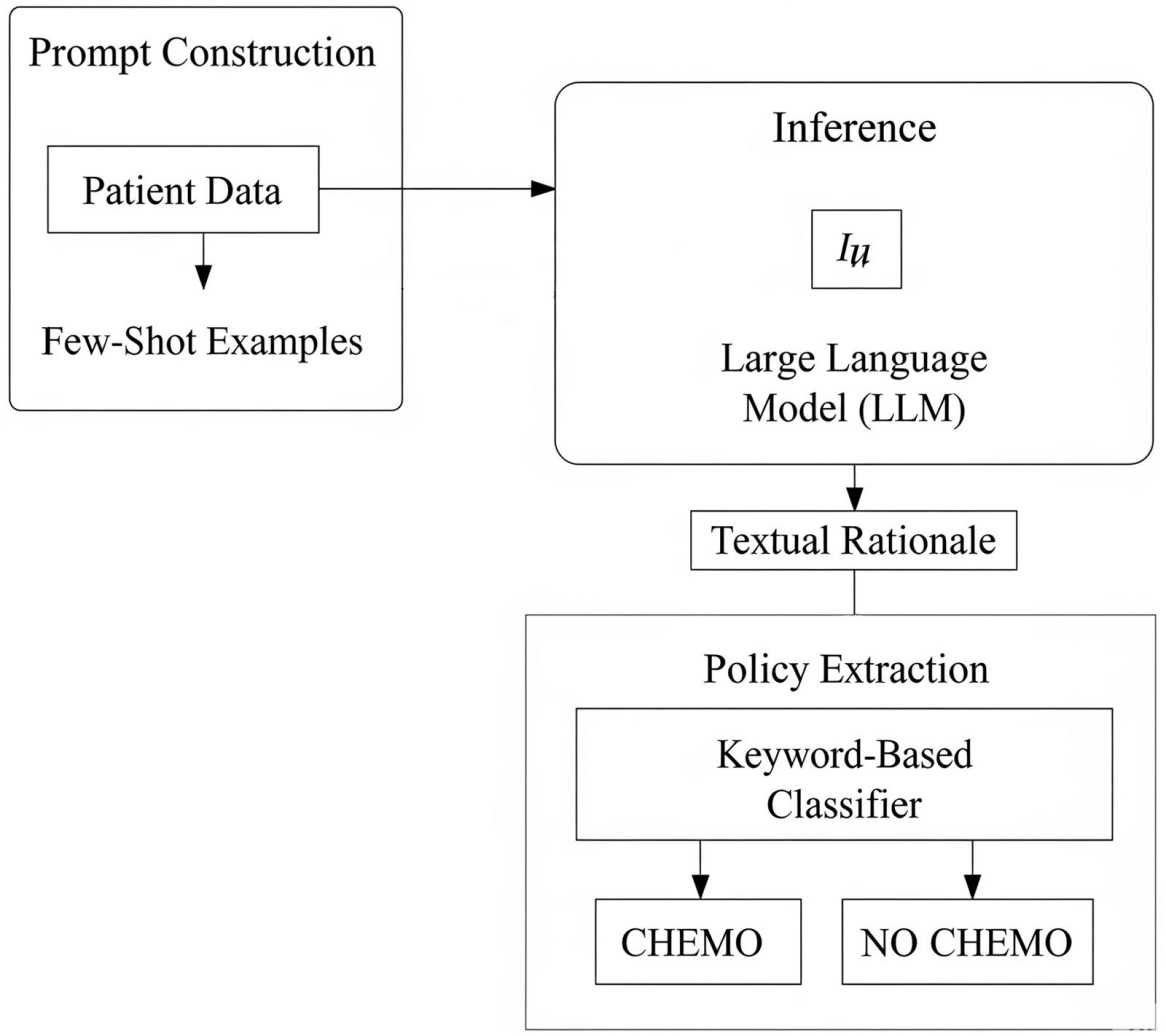
Architectural schematic of the prompt construction and few-shot GPT-4 framework for deriving chemotherapy recommendations.

We constructed the prompt template by sequentially concatenating several structured input-output instances, each of which matched a clinically relevant feature profile with an expert-style textual justification, in order to facilitate efficient few-shot generalisation. The purpose of this series of exemplars is to subtly condition the LLM on the desired structure and content of reasoning. In order to enable inference-time reasoning based on the given clinical context, the prompt concludes with a query instance that simply includes the patient’s features and a placeholder for the model to construct a relevant explanation, Equation 7. The overall pipeline for constructing prompts and deriving rationale-driven chemotherapy decisions with GPT-4 is illustrated in Figure 2.

$$\begin{array}{rl} \text{Prompt} & =\sum_{i=1}^{k}[{\text{Features}}_{i}\Rightarrow {\text{Rationale}}_{i}]\\ & \;+{\text{Target Features}}_{k+1}\Rightarrow ? \end{array}$$ (7)

Here, each example i∈[1,k] represents a real or synthetic patient scenario consisting of: a tabularized feature summary (e.g., “ER+: Yes, HER2: Negative, Stage: III”), and a corresponding rationale (e.g., “ER positivity supports endocrine therapy; high-risk stage warrants chemotherapy.”). The final query instance ${\text{Target Features}}_{k+1}$ prompts the LLM to generate a rationale without seeing the ground truth decision, mimicking few-shot inference. For certain deterministic results, we utilised OpenAI’s GPT-4 with temperature = 0 and presented data in tabular way that clinicians could understand, for instance shown in Table 3:

**Table 3:** Case-based example of chemotherapy decision.

Item	Description
Patient Profile:	ER: Positive; PR: Negative; HER2: 2+; Grade: 3; Nodes: 3; Tumor Size: 4.1 cm
Question:	Should chemotherapy be considered? Why?
Answer:	High tumor grade and nodal involvement suggest high-risk features; chemotherapy is indicated despite ER positivity.

#### 3.5.2 Postprocessing and policy extraction

After the generated rationales were analysed, an implicit binary recommendation, CHEMO or NO CHEMO, was extracted from the generated text. To operationalize the textual rationale into a structured recommendation, we employed a lightweight rule-based postprocessing approach using clinically relevant keywords and treatment-related phrases. This strategy was selected to maintain interpretability and deterministic mapping between rationale outputs and treatment categories within the exploratory framework of this study. Although this approach enabled straightforward extraction of treatment recommendations, rule-based parsing may oversimplify nuanced clinical language and may not fully capture contextual semantics or uncertainty expressed in the generated rationale. Therefore, the extracted policy outputs should be interpreted as approximate representations of GPT-4 reasoning rather than definitive clinical recommendations.

- “favor chemotherapy”, “high-risk”, “triple-negative” → CHEMO
- “support endocrine therapy”, “low-risk” → NO CHEMO

This allowed us to establish a binary treatment suggestion strategy based on the logic produced by the LLM. In particular, we refer to the decision function obtained from LLM as:

$$\text{LLM}(x)=\left\{ \begin{array}{cc} 1,\; & \text{if rationale implies chemotherapy}\;\\ 0,\; & \text{otherwise}\; \end{array}\right.$$

Without the use of an explicit classification head, the binary chemotherapeutic prescription deduced from the LLM-generated justification for input x is represented here by ${\hat{y}}_{\text{LLM}}(x)$. The ML-based suggestion ${\hat{y}}_{\text{ML}}(x)$ and the actual treatment choices documented in the dataset were then directly compared to this policy.

#### 3.5.3 Inference settings and evaluation strategy

LLM inference was performed using the OpenAI GPT-4 API (version 2023-08-16). To ensure reproducibility, we used deterministic decoding by setting temperature = 0 and turning off nucleus sampling. This approach made sure that the same prompts produced the same recommendations. Each query, which included 5 examples plus a test patient, resulted in an output of about 100 to 150 tokens. The average time for inference was 4.1 s, with a deviation of 0.8 s per patient. Using the API costs an average of 0.00 per patient for prompt and completion tokens since we utilized the free version. This computational demand is very small compared to regular genomic tests.

### 3.6 Model explainability using SHAP

To improve interpretability and provide feature-level explanations for the Random Forest predictions, we incorporated SHapley Additive exPlanations (SHAP) analysis. SHAP is a game-theoretic explainability framework that estimates the contribution of each feature to an individual prediction while preserving local accuracy and consistency. In clinical decision-support settings, SHAP enables transparent interpretation of complex machine learning models by quantifying how specific clinicopathologic variables influence chemotherapy recommendations.

We used the TreeExplainer implementation for Random Forest models to compute SHAP values on the held-out test set. Global feature importance was assessed using the mean absolute SHAP values across all samples, while summary plots were used to visualize both the magnitude and direction of feature contributions. Positive SHAP values indicate an increased likelihood of chemotherapy recommendation, whereas negative values indicate a reduced likelihood. This analysis complements permutation importance and provides an additional explainability layer aligned with clinically interpretable reasoning.

### 3.7 Evaluation strategy

The performance of both the ML and LLM components was evaluated using standard metrics that match clinical interpretation. The Random Forest was assessed based on classification performance. In contrast, GPT-4, accessed through the OpenAI API, was evaluated according to its agreement with clinician-written rationales, its contribution to policy value, and its clarity of explanation. This modest agreement was expected. GPT-4 was not designed to provide definitive clinical rationales. Its role in this framework is interpretive; it highlights relevant covariates and offers narrative explanations that complement, but do not replace, expert oncological reasoning.

#### 3.7.1 Evaluation of the random forest model

The Random Forest model gives probabilistic predictions $\hat{\;p}(x)$ for chemotherapy recommendations based on a feature vector x. To change this probability into a binary decision, we use the threshold τ (as described in Section 2), resulting in the final prediction $\hat{y}(x)$. For evaluation, we built the confusion matrix as, Equation 8:

$$C=\left[\begin{array}{cc} \text{TP} & \text{FP}\\ \text{FN} & \text{TN} \end{array}\right]$$ (8)

where TP, FP, FN, and TN represent true positives, false positives, false negatives, and true negatives, respectively. From this, we computed accuracy, precision, recall, and the $F_{1}$-score. These metrics highlight important clinical trade-offs. Recall shows how well we can identify patients needing chemotherapy, which is crucial in oncology. Precision measures how well we avoid giving unnecessary treatment. To summarize discrimination across thresholds, we report the Area Under the Receiver Operating Characteristic Curve (AUC-ROC), Equation 9:

$$\text{AUC}=\int_{0}^{1}\text{TPR}\;\left({\text{FPR}}^{-1}(u)\right)\;du$$ (9)

where TPR is the true positive rate and FPR is the false positive rate. AUC offers a measure of separability without a specific threshold. This helps ensure the model is not too focused on one operating point. Together, the confusion matrix and AUC provide both operational and overall views of predictive power.

#### 3.7.2 Evaluation of the LLMs (GPT-4)

Unlike supervised classifiers, GPT-4 generates textual rationales that form the basis for binary treatment policies. The decision function is formalized as, Equation 10:

$${\hat{y}}^{\text{GPT-4}}(x)=\left\{ \begin{array}{cc} 1,\; & \text{if the rationale implies chemotherapy},\;\\ 0,\; & \text{otherwise}.\; \end{array}\right.$$ (10)

To evaluate this policy, we compared GPT-4-derived recommendations with both the Random Forest policy and actual treatment assignments. We measured policy effectiveness using inverse probability weighted (IPW) policy value (44), which indicates the expected survival gain based on the recommendations, Equation 11:

$$V(\pi )=\frac{1}{N}\sum_{i=1}^{N}\frac{1\{\pi (x_{i})=T_{i}\}\cdot Y_{i}}{e(T_{i}|x_{i})}$$ (11)

where $\pi (x_{i})$ is the policy’s recommendation, $T_{i}$ is the actual treatment received, $Y_{i}$ is the observed outcome (alive at 5 years), and $e(T_{i}|x_{i})$ is the estimated treatment likelihood. This directly tests whether GPT-4 rationales lead to better survival benefits than “treat-all” or “treat-none” methods. Finally, we conducted Mann–Whitney U-tests to compare uplift distributions between patients who had GPT-4 recommend chemotherapy and those who did not. This statistical check confirms that GPT-4’s decisions relate to significant survival differences instead of being randomly assigned.

### 3.8 Experimental setup and reproducibility

To ensure transparency and reproducibility, we document the full experimental setup, hardware, and parameterization used in this study. Table 4 provides a summary of the inference configuration, dataset partitioning, program dependencies, and computational setup. The machine learning and LLM-based components can be replicated under similar conditions by independent researchers thanks to these specifications.

**Table 4:** Experimental setup for reproducibility of the study.

Category	Specification	Notes
Operating System	Ubuntu 22.04 LTS	64-bit, Linux kernel 5.15
Python Version	Python 3.11	Virtual environment with pip
Libraries	scikit-learn 1.4, lifelines 0.28	For ML and survival analysis
	OpenAI GPT-4 API (Aug 2023)	For LLM inference
Hardware	Intel i7-12700 CPU	12 cores, 2.1 GHz
	32 GB RAM	DDR4
	NVIDIA RTX 3,060	12 GB VRAM, CUDA 11.7
Data Split	70% train/15% validation/15% test	Stratified by chemotherapy label
Hyperparameters	Grid search on validation set	Random seeds fixed for reproducibility
Survival Model	Logistic regression (propensity)	Inverse probability weighting (IPTW)
	Cox proportional hazards	Covariate balance via SMD
LLM Inference	GPT-4, temperature = 0	Deterministic decoding, no nucleus sampling
	Prompt length: 5-shot	600–800 tokens including rationale

## 4 Experimental analysis

### 4.1 Random forest performance

The Random Forest model showed strong predictive performance in simulating chemotherapy decisions based on pre-treatment characteristics. As shown in Table 5, the model achieved an overall accuracy of 90% and clearly distinguished between treated and untreated patients. Specifically, the classifier attained a precision of 0.92 and a recall of 0.95 for non-chemotherapy cases. For patients receiving chemotherapy, the recall was 0.68 and the F1-score was 0.73. This imbalance reflects the inherent difficulty of detecting the minority chemotherapy group; however, the model maintains clinically meaningful sensitivity (recall = 0.68), supported by cost-sensitive training and threshold optimisation strategies designed to prioritise minority-class detection.

**Table 5:** Classification report of Random Forest model on chemotherapy decision-making.

Class	Precision	Recall	F1-Score
0.0	0.92	0.95	0.94
1.0	0.79	0.68	0.73
Macro Avg	0.85	0.82	0.83
Weighted Avg	0.89	0.90	0.89
Accuracy = 0.90			

**Figure 3:**
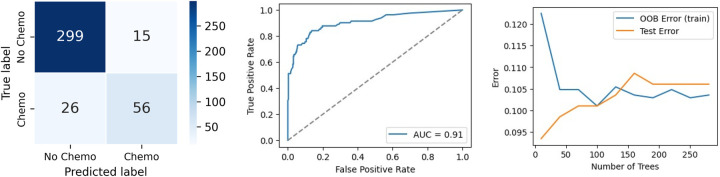
Performance of Random Forest model: From left side, Confusion matrix on the test set, ROC curve with AUC = 0.91, and OOB and test error across trees.

Table 5 summarizes the classification report. Figure 3 shows three complementary perspectives on model behavior. The receiver operating characteristic (ROC) curve, shown in Figure 3, has an AUC of 0.91. This confirms strong global separability between chemotherapy and no-chemotherapy recommendations. The out-of-bag (OOB) error and test error curves, illustrated in Figure 3, converge as the number of trees increases. This demonstrates stability and shows there is no overfitting. Finally, the confusion matrix in Figure 3 highlights the balanced predictive ability of the model. Most errors occur in the minority chemotherapy-positive group.

These results show that the Random Forest model effectively captures nonlinear interactions among tumor size, nodal involvement, histologic grade, and receptor status. The strong performance means that the model can reproduce clinically important treatment patterns. It provides a reliable view of chemotherapy decisions based on pre-treatment factors.

### 4.2 Propensity diagnostics and causal survival uplift analysis

**Figure 4:**
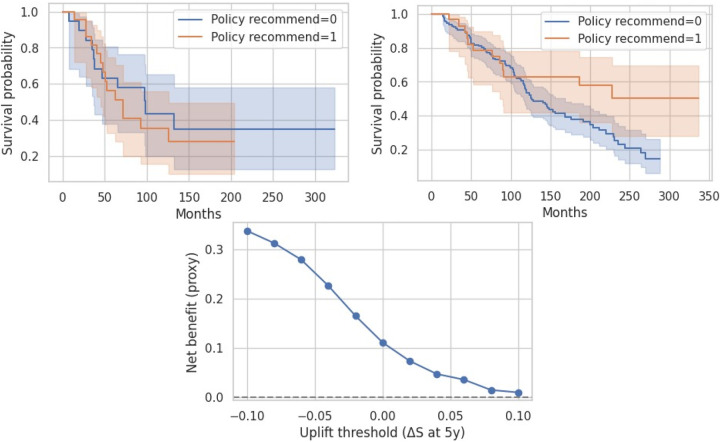
Kaplan-Meier survival categorised by treatment status and model policy. From left side, Chemotherapy-receiving patients, categorised by policy recommendation; Chemotherapy-non-receiving patients, categorised by policy recommendation.

To estimate individualized treatment effects, we used inverse probability weighting (IPW) with Cox proportional hazards models stratified by chemotherapy status. The fitted uplift model achieved a propensity score AUC of 0.916. This shows that pre-treatment factors effectively separated the treated and untreated groups. Figure 4 summarizes the causal survival results. Figures 4 show Kaplan–Meier survival curves for patients who received chemotherapy and those who did not, each divided by the model’s policy recommendation. While uncertainty bands are wide due to the small subgroup size, we see clear separation.

**Figure 5:**
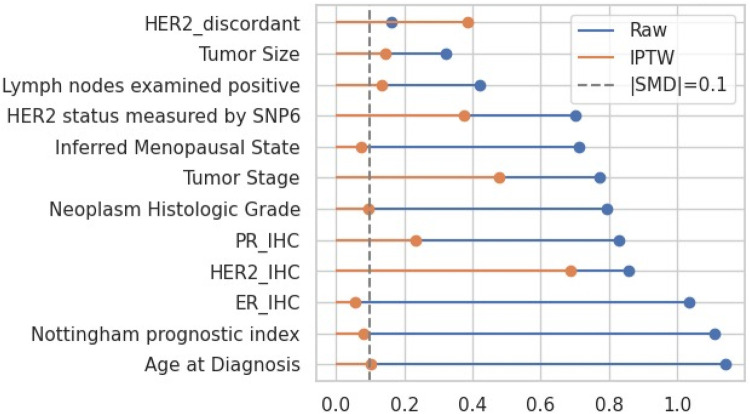
Covariate balance between treatment groups before and after IPTW.

Table 6 shows the uplift calibration in quintiles along with summary statistics. The mean uplift at 5 years rate ($\Delta S_{5y}$) was −0.062, and the median was −0.052. This reflects variability in treatment benefit among patients. Notably, about 25.8% of patients were recommended chemotherapy under the policy (uplift >0), showing that the approach targeted specific individuals rather than applying universal treatment. To validate the causal framework, we checked the balance between treated and untreated groups using standardized mean differences (SMD). Figure 5 shows that raw covariates had significant imbalance, especially for ER, HER2, tumor stage, and menopausal status. After applying inverse probability of treatment weighting (IPTW), the SMD values were greatly reduced, with most features nearing the accepted threshold of 0.1. This suggests that weighting successfully reduced confounding and supports the trustworthiness of the following uplift survival estimates.

**Table 6:** Calibration and summary of uplift analysis.

Uplift bin (ΔS_5y)	n	Mean uplift	Alive 5y_rate	Chemo_rate
(−0.549, −0.236	0.225	0.050
(−0.137, −0.0769)	39	−0.104	0.231	0.051
(−0.0769, −0.0325)	40	−0.052	0.400	0.225
(−0.0325, 0.0119)	39	−0.010	0.513	0.205
(0.0119, 0.628)	40	0.091	0.475	0.500

#### 4.2.1 Propensity diagnostics

**Figure 6:**
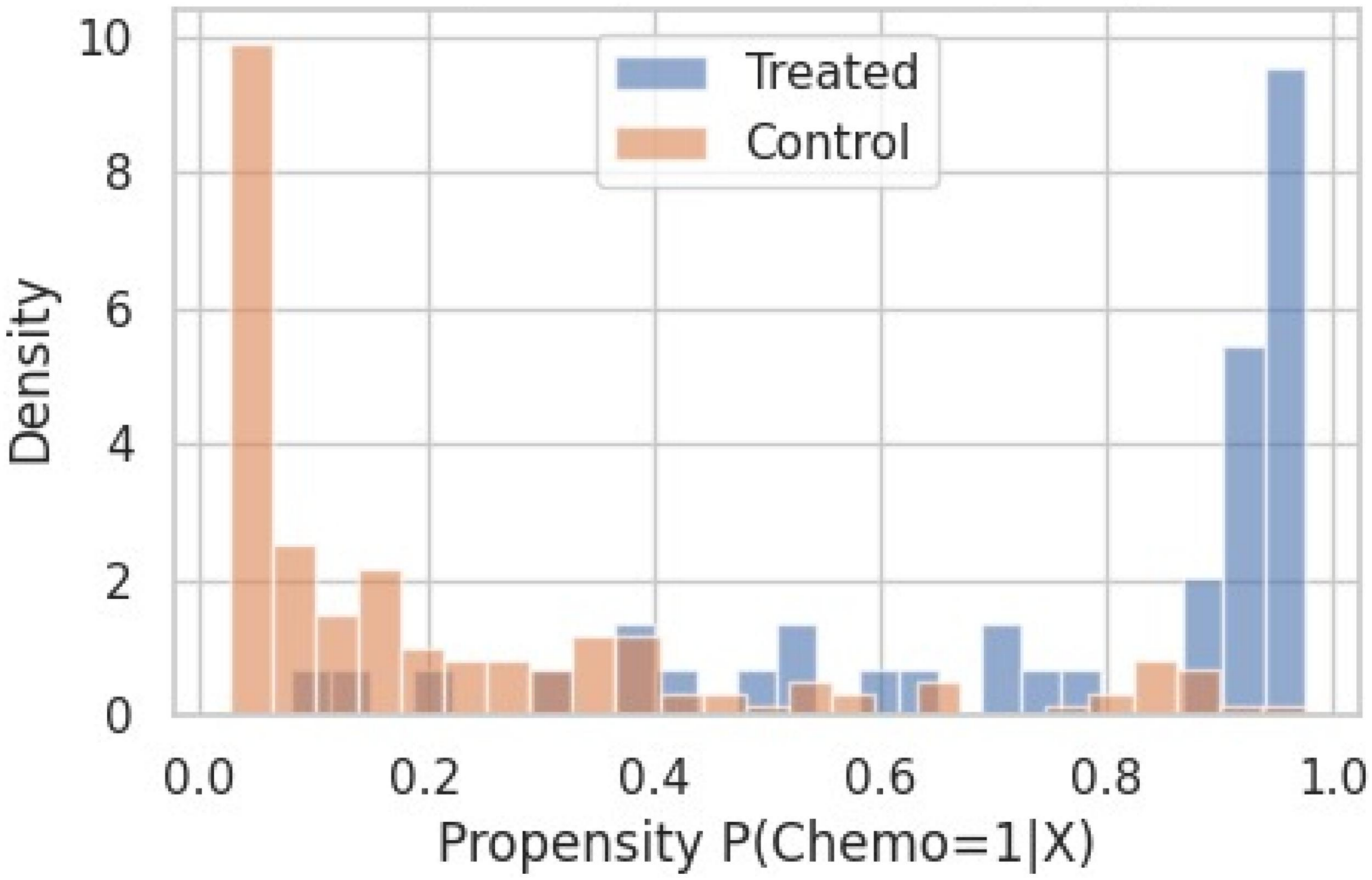
Propensity score overlap in the test cohort.

Before estimating the benefit of individualized treatment, we checked if inverse probability weighting (IPW) achieved adequate balance between the chemotherapy-treated and untreated groups. Figure 6 shows the overlap of propensity scores. Treated patients clustered near P(T=1∣X)≈1, while controls were near 0. After applying the weights, the standardized mean differences (SMDs) improved significantly. Table 7 summarizes the balance metrics for the main covariates. Initial imbalances exceeding |SMD|>1.0 (such as age, Nottingham Prognostic Index, and ER status) dropped below 0.1 after IPTW, meeting standard balance thresholds for most covariates.

**Table 7:** Standardized mean differences before and after IPTW weighting.

Feature	SMD (Raw)	SMD (IPTW)
Age at diagnosis	−1.141	−0.100
Nottingham Prognostic Index	1.109	0.079
ER (IHC)	−1.035	0.055
PR (IHC)	−0.830	−0.233
HER2 (IHC)	0.856	0.685
HER2 (SNP6)	−0.701	−0.375
HER2 discordant	−0.161	−0.386
Tumor Size	0.321	0.146
Tumor Stage	0.771	0.479
Lymph Nodes Positive	0.421	−0.135
Histologic Grade	0.793	−0.096
Menopausal State	0.711	−0.075

However, HER2-related variables remained substantially imbalanced after weighting (HER2-IHC SMD = 0.685; HER2-SNP6 SMD =−0.375). This residual imbalance reflects the near-deterministic treatment assignment in HER2-positive patients within the METABRIC cohort, where the majority receive chemotherapy (often alongside targeted therapy). As a result, the positivity assumption underlying IPTW is partially violated for this subgroup, leading to extreme propensity scores and unstable weights. Consequently, individualized uplift estimates for HER2-positive patients should be interpreted with caution, as they may not reliably reflect counterfactual treatment effects. To assess the impact of this limitation, a sensitivity analysis excluding HER2-positive patients was conducted, yielding qualitatively consistent policy value estimates (0.398 vs. 0.418 overall). This suggests that the primary findings are not solely driven by HER2-associated imbalance, although the limitation remains important for subgroup interpretation.**

#### 4.2.2 Survival uplift results

Weighted Cox models were fitted separately for the treated and control groups to estimate 5-year survival under different scenarios. Hazard ratio (HR) summaries are in Table 8. In the chemotherapy group, age (HR = 1.05, 95% CI 1.03, 1.06), nodal positivity (HR = 1.06, 95% CI 1.04, 1.09), and histologic grade (HR = 1.51, 95% CI 1.15, 2.00) were identified as independent negative factors. In the control group, tumor size (HR = 1.02, 95% CI 1.01, 1.03), Nottingham prognostic index (HR = 1.28, 95% CI 1.03, 1.59), and positivity for HER2 (HR = 1.87, 95% CI 1.18, 2.98) were strongly related to lower survival rates. These findings support the biological reasonableness of the model outputs.

**Table 8:** Robust Cox proportional hazards models with IPTW weighting.

Covariate	HR (treated)	95% CI	p	HR (control)	95% CI	p
Age at Diagnosis	1.048	1.033–1.063	<0.001	1.023	1.007–1.040	0.006
Menopausal State	1.418	0.988–2.035	0.058	1.566	1.062–2.311	0.024
Tumor Size	1.000	0.993–1.007	0.962	1.016	1.007–1.025	<0.001
Tumor Stage	0.980	0.629–1.525	0.927	0.887	0.637–1.237	0.481
Lymph Nodes Positive	1.063	1.036–1.091	<0.001	1.043	1.007–1.081	0.019
Nottingham Prognostic Index	0.927	0.750–1.146	0.485	1.278	1.027–1.589	0.028
Histologic Grade	1.514	1.147–1.997	0.003	0.908	0.678–1.216	0.518
ER (IHC)	0.996	0.687–1.444	0.984	1.006	0.648–1.563	0.978
PR (IHC)	0.756	0.525–1.088	0.132	0.761	0.555–1.045	0.091
HER2 (IHC)	1.329	0.941–1.878	0.106	1.872	1.175–2.981	0.008
HER2 (SNP6)	0.971	0.828–1.140	0.720	1.105	0.934–1.307	0.244
HER2 discordant	1.122	0.646–1.947	0.683	1.220	0.853–1.745	0.275

**Figure 7:**
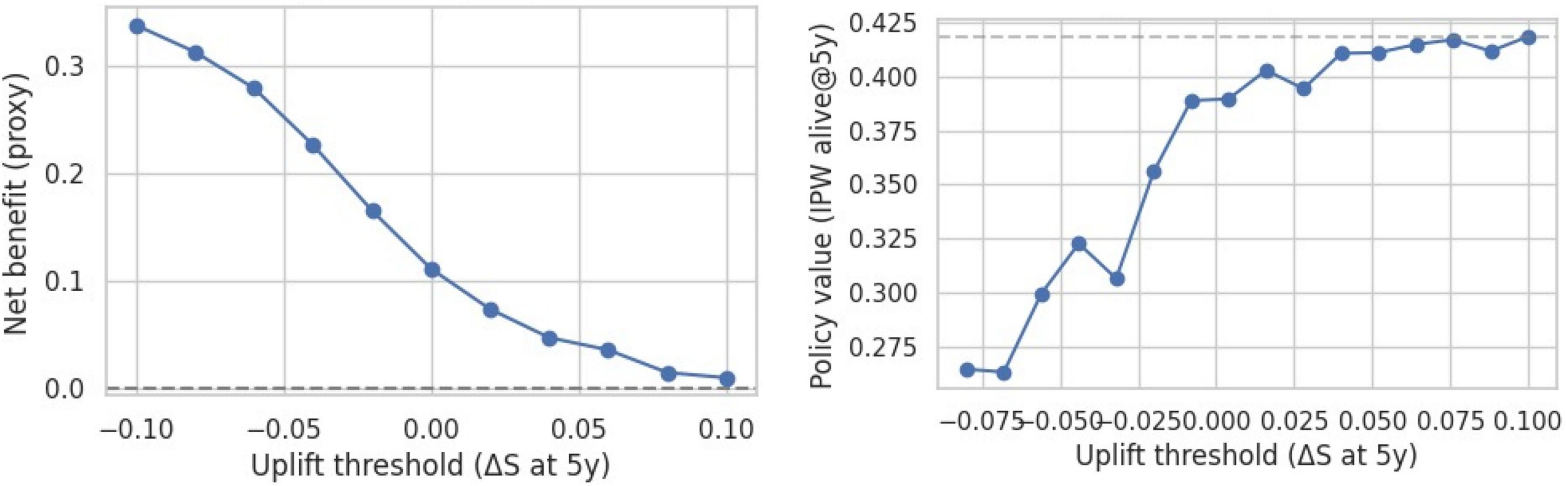
From left side, net benefit curves across uplift thresholds, quantifying trade-offs between overtreatment and undertreatment. IPW-estimated policy value at 5 years as a function of uplift threshold, showing maximum benefit around τ=0.08.

Survival stratification by policy recommendation was not statistically significant among chemotherapy recipients (log-rank p=0.59). In the non-chemotherapy group, a trend toward significance was observed (log-rank p=0.059), suggesting potential separation that warrants further validation in larger cohorts. This suggests that uplift-based policies can capture differences more effectively among untreated patients. Figure 7 shows that the net clinical benefit decreases as the rising thresholds become stricter. This change leads to less overtreatment but more false negatives. In contrast, Figure 7 presents IPW policy values. It shows a plateau of benefit across thresholds between 0.05 and 0.10, indicating strong treatment guidance in this range. The maximum value occurred around τ=0.08. Clinically, this suggests that only a subset of biologically high-risk patients may derive measurable survival benefit from chemotherapy, supporting treatment de-escalation in lower-risk populations. Bootstrap estimates yielded a policy value of 0.387 (95% CI 0.276 to 0.496) and a recommended treatment share of 25.8% (95% CI 19.7 to 31.8). Finally, Table 9 presents subgroup analyses based on ER and HER2 status. Patients with ER- tumors showed the highest benefit, with a policy value of 0.600. In contrast, the ER + and HER2- subgroups had a lower mean increase, suggesting that the benefit of chemotherapy is more concentrated in high-risk biological groups.

**Table 9:** Subgroup policy evaluation (IPW, 5-year outcomes).

Subgroup	n	Policy value	Mean uplift
ER+	153	0.322	−0.077
ER-	40	0.600	0.014
HER2+	32	0.456	−0.003
HER2-	166	0.375	−0.074

### 4.3 LLM (GPT-4) policy results

**Figure 8:**
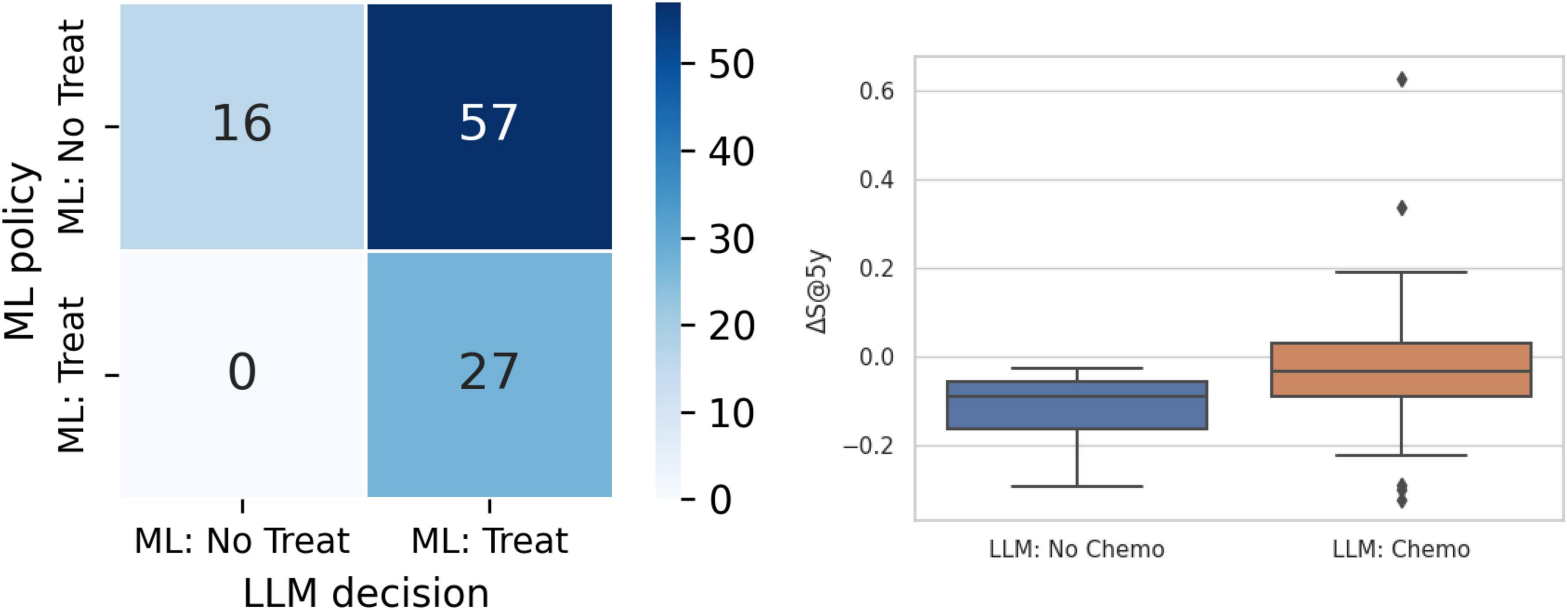
GPT-4 policy evaluation. From the left side, agreement matrix vs. random forest policy. Uplift distribution stratified by GPT-4 decision.

The GPT-4 derived policy showed moderate agreement with the Random Forest baseline, with an accuracy of 0.430 and Cohen’s κ of 0.132 (95% CI: 0.068 to 0.202). As shown in Figure 8, computed on 100 held-out test cases, most disagreements happened when GPT-4 suggested chemotherapy while the machine learning policy did not. This indicates a tendency towards recommending more aggressive treatment. The uplift distributions confirmed the ability to stratify, with a Mann-Whitney U of 335.0 and a p value of 0.0016. Patients recommended for chemotherapy by GPT-4 had higher benefit estimates, Figure 8.

Comparisons at the policy level, as shown in Table 10, revealed that the GPT-4 policy ranked below the machine learning uplift-based recommendations, but above the treat-none option. This suggests it has some clinical utility. Notably, Treat-All achieved the highest survival value, while hybrid rules matched the performance of the machine learning policy without exceeding it. Finally, recommendation cards outline patient-level reasoning. For example, GPT-4 correctly withheld chemotherapy in an ER-positive case (Patient 404: uplift -0.076, rationale: “*ER positivity supports endocrine therapy*”). However, it often over-recommended treatment in uncertain situations (Patient 515: uplift -0.177, ML: no chemo, GPT-4: chemo, rationale: *triple-negative; high-risk features favor chemotherapy*). Although the overall agreement between the ML and GPT-4 policies was moderate (accuracy 0.43, κ=0.13), the strength of GPT-4 lies in its ability to provide rational recommendations. To illustrate this, Table 11 shows cases where the two policies differed. Each row includes the prediction of ML, the decision of GPT-4, the actual treatment assigned, and the rationale given by GPT-4. These examples show that GPT-4 often justified chemotherapy for reasons such as endocrine ineligibility, HER2 positivity, or high-risk pathological features. This led to more aggressive recommendations than those from the RF model. These cases demonstrate both the clarity and clinical validity of the rationales generated by LLMs.

**Table 10:** Policy value comparison (IPW alive @ 5y).

Policy	Value
Treat-All	0.436
ML policy	0.418
Hybrid AND	0.418
GPT-4 only	0.383
Hybrid OR	0.383
Treat-None	0.379

**Table 11:** Representative patient-level decisions where GPT-4 and ML policies diverged.

Patient ID	ML Policy	GPT-4 Policy	Actual Chemo	Rationale (LLM output, condensed)
202	No Chemo	Chemo	No	ER positivity supports endocrine therapy; high-risk tumor size favors chemo
1427	No Chemo	Chemo	No	Triple-negative profile; endocrine ineligible; chemotherapy recommended
1790	No Chemo	Chemo	No	HER2 positivity/genomic gain favors anti-HER2 + chemotherapy
1759	No Chemo	Chemo	Yes	Triple-negative pattern with nodal burden; chemotherapy appropriate

### 4.4 Baseline comparison

To validate the robustness of the proposed RF framework, we compared its performance against five common classifiers: Logistic Regression (LR), k-Nearest Neighbors (KNN), Support Vector Machine (SVM), Gradient Boosting (GB), and a feed-forward neural network (NN). Table 12 shows the results in terms of accuracy, precision, recall, and F1-score on the held-out test set. Overall, the accuracy ranged from 0.85 to 0.89, with varying recall and precision trade-offs. For the positive class (patients requiring chemotherapy), SVM and logistic regression had low recall but high precision, which could result in undertreatment in clinical settings. Although KNN and Gradient Boosting provided a better balance, their recall was still quite low. Due to its limited training data, the neural network baseline exhibited the weakest generalization. In contrast, the Random Forest achieved the highest accuracy (0.90) while keeping precision (0.89) and recall (0.82) balanced. This shows its capacity to capture nonlinear interactions without losing generalization. Importantly, the higher recall compared to linear models reflects better sensitivity in detecting patients eligible for chemotherapy, which is crucial in clinical care. This comparison establishes Random Forest as the most dependable choice for further causal survival and LLM-based analyses.

**Table 12:** Performance comparison with baseline classifiers.

Model	Accuracy	Precision	Recall	F1-score
LR	0.86	0.88	0.86	0.87
KNN	0.89	0.88	0.89	0.88
SVM	0.86	0.88	0.86	0.87
GB	0.89	0.88	0.89	0.88
NN	0.85	0.85	0.85	0.85
RF (ours)	0.90	0.89	0.90	0.89

**Figure 9:**
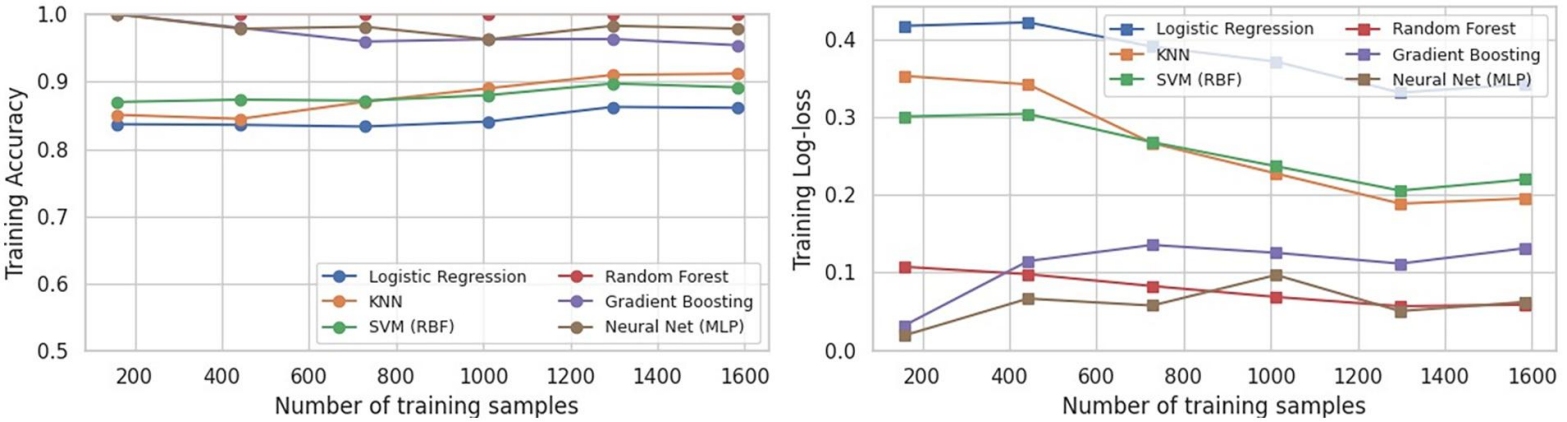
Training dynamics across all baseline models: From left to right, training accuracy as a function of sample size and training log loss as a function of sample size.

Figure 9 gives more insight into how the model behaves as the training set size increases. In panel (a), both Random Forest and Gradient Boosting showed consistently high training accuracy, indicating they can capture nonlinear relationships. However, in panel (b), the Random Forest had a lower log loss, suggesting that it provides better probability estimates than gradient boosting. The neural network reached nearly perfect training accuracy but had unstable log-loss, which is a sign of overfitting due to the limited dataset size. In contrast, logistic regression and SVM had flatter learning curves, showing little improvement after 1,000 samples, which reflects the limitations of linear decision boundaries. KNN showed some improvement with a larger training size, but reached a plateau in both accuracy and loss. Overall, these behaviors show that Random Forest strikes the best balance between predictive accuracy and probability calibration, making it the preferred choice for the upcoming causal uplift and LLM integration.

#### 4.4.1 Error analysis

**Figure 10:**
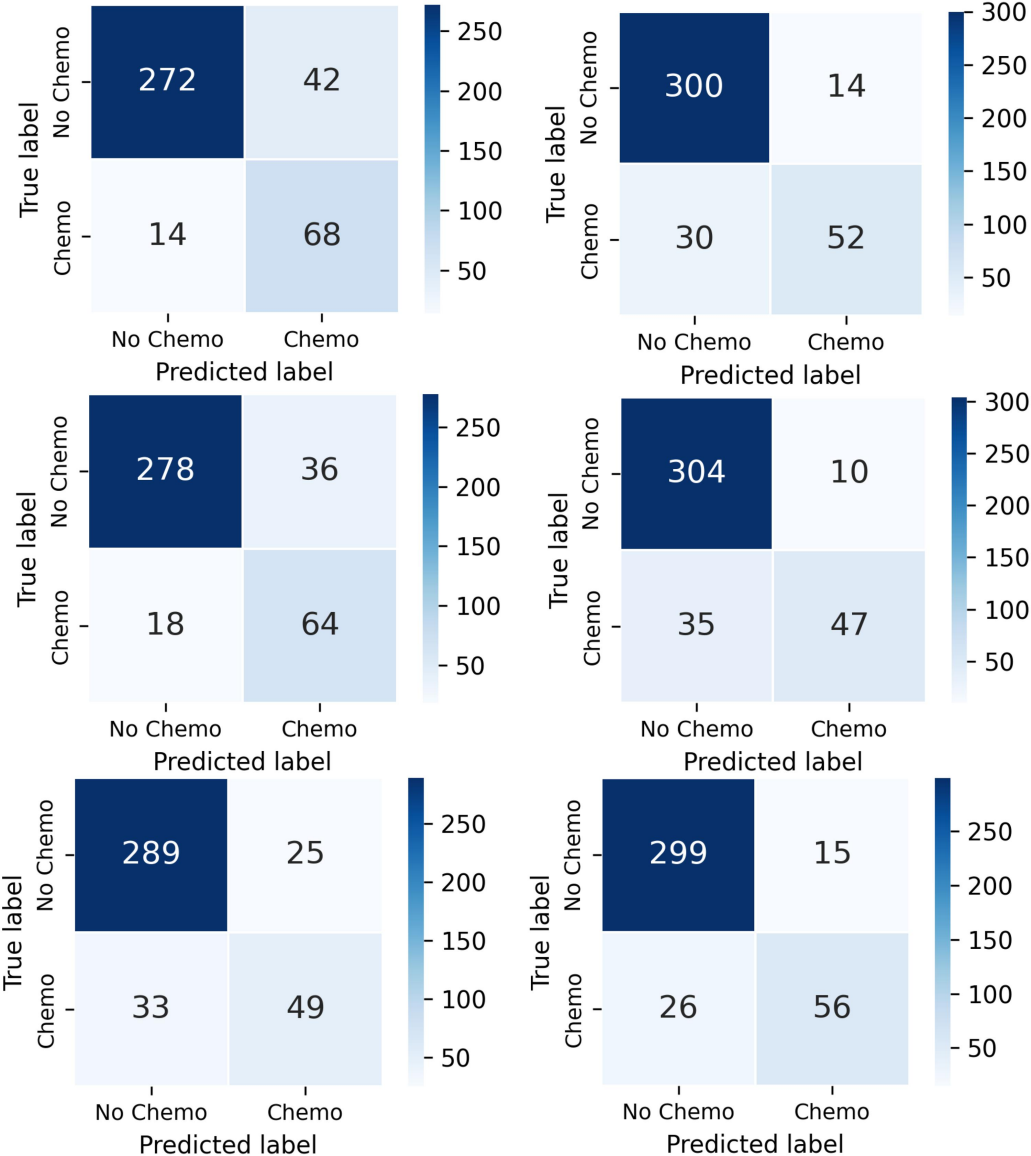
Baseline comparison confusion matrix: From left to right, LR, KNN, SVM, GB, NN, RF.

To better understand the differences among the models, we looked at the confusion matrices shown in Figure 10. This review points out the types of mistakes made by each classifier and their effects on clinical decision-making. For logistic regression and SVM, Figures 10, false negatives were the primary error type. Several patients eligible for chemotherapy were wrongly classified as not needing it. While these models achieved high precision, their limited recall poses a risk of undertreatment, which could have serious consequences in oncology practice. KNN and Gradient Boosting Figures 10 showed more balanced error patterns. However, both still produced false negatives at a significant rate, indicating that neighborhood-based or boosting methods may not fully account for variations in receptor status or nodal burden. The neural network baseline Figure 10 experienced both false positives and false negatives. This aligns with overfitting and unstable generalization due to the limited size of the cohort. It highlights the challenge of using deep learning methods on relatively small clinical datasets without regularization or pretraining. On the other hand, the Random Forest Figure 10 struck the best balance between false positives and false negatives. Although there were a few predictions of overtreatment (false positives), the model showed a significantly higher recall for the positive class, reducing the risk of undertreatment. Clinically, this trade-off is preferable, as missing a patient who needs chemotherapy is more harmful than misrecommending treatment. Finally, this error analysis shows that the Random Forest lowers the crucial false negative errors compared to other models. Its reliability supports its role as the basis for causal survival analysis and integration of GPT-4-based rationale in the following sections.

### 4.5 SHAP-based explainability analysis

**Figure 11:**
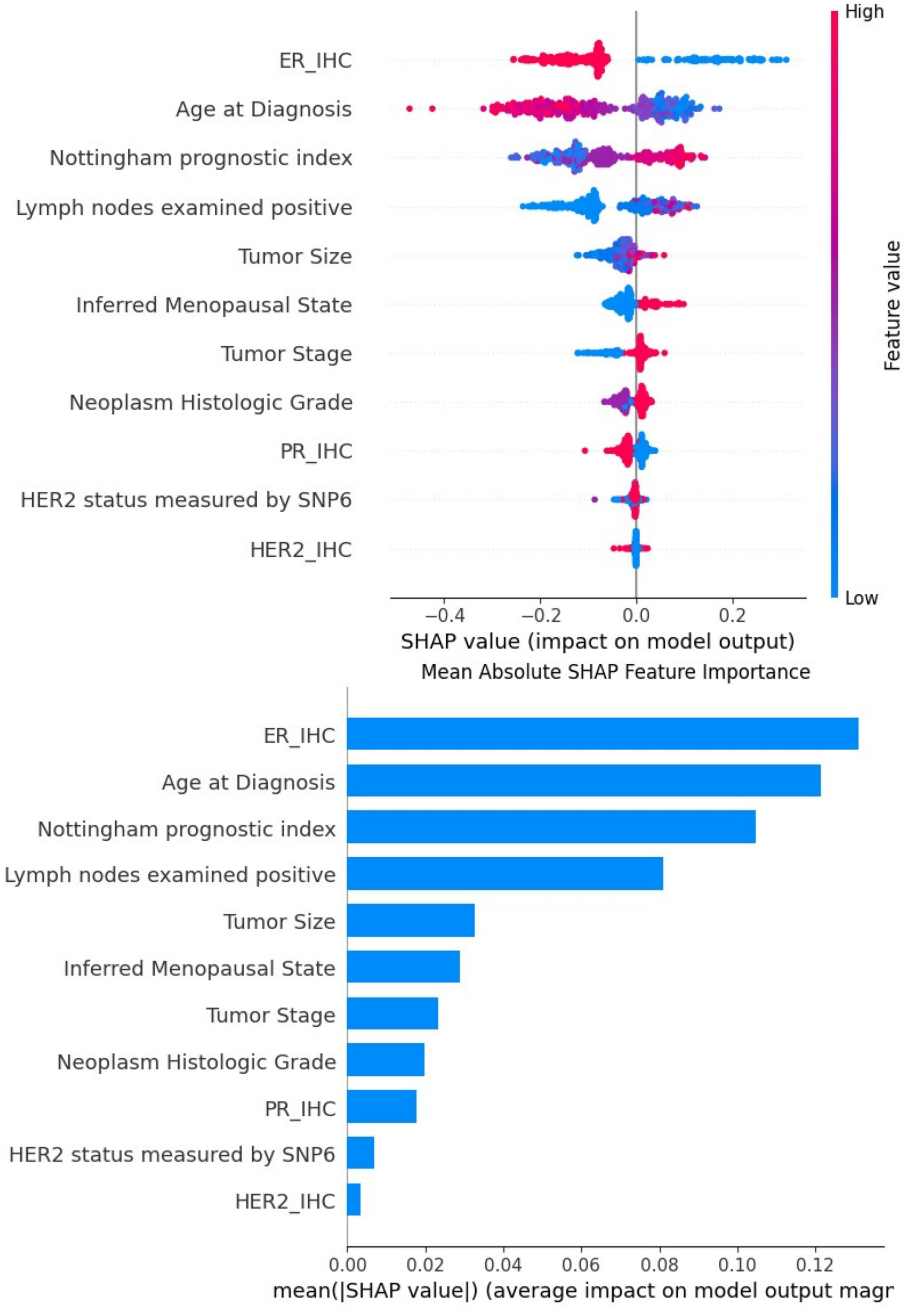
SHAP-based explainability analysis of the Random Forest chemotherapy prediction model. SHAP summary plot showing feature contribution magnitude and direction across patients. Mean absolute SHAP values representing global feature importance rankings.

To further interpret the Random Forest predictions, we performed SHAP analysis on the held-out test cohort. Figure 11 illustrates the SHAP summary and mean absolute importance plots for the chemotherapy prediction model. The results showed that ER status measured by IHC was the most influential feature, followed by age at diagnosis, Nottingham prognostic index (NPI), lymph node positivity, and tumor size. These findings are clinically consistent with established breast cancer treatment decision factors.

The SHAP summary plot demonstrated both the direction and magnitude of feature contributions. Negative ER status and higher nodal burden were generally associated with increased chemotherapy recommendation probability, whereas positive ER status contributed toward reduced chemotherapy likelihood, reflecting the clinical preference for endocrine therapy in hormone receptor–positive disease. Similarly, higher NPI scores, larger tumor size, and advanced tumor stage shifted model predictions toward chemotherapy recommendation. Importantly, the SHAP-derived rankings were consistent with the permutation importance and causal uplift analyses presented earlier, supporting the biological plausibility and internal consistency of the framework. These results suggest that the Random Forest model learned clinically meaningful relationships rather than relying on spurious correlations. The integration of SHAP therefore improves transparency and strengthens interpretability of the machine learning component within the proposed decision-support pipeline.

## 5 Discussion

The proposed study explored the integration of predictive machine learning, causal uplift modeling, and GPT-4-based rationale generation for personalized chemotherapy decision support in breast cancer. The Random Forest model achieved strong predictive performance (AUC=0.91), suggesting that chemotherapy decisions are influenced by complex interactions among clinicopathologic variables rather than simple linear relationships. Across the predictive and explainability analyses, ER status, nodal involvement, tumor size, and Nottingham Prognostic Index emerged as the most influential factors, which is consistent with established breast cancer risk stratification principles. The uplift analysis further showed that chemotherapy benefit was not uniformly distributed across patients, with ER-negative and node-positive subgroups demonstrating greater estimated benefit. These findings support the importance of individualized treatment estimation rather than uniform treatment allocation. Despite the encouraging performance, several clinically important trade-offs remained evident. The chemotherapy-positive recall of 0.68 indicates that some potentially eligible patients were not identified, highlighting the difficulty of balancing overtreatment reduction against the risk of undertreatment in oncology decision-support systems. Similarly, although the uplift-guided policy reduced chemotherapy recommendations substantially while maintaining survival outcomes relatively close to the treat-all strategy, these findings should be interpreted cautiously because observational datasets such as METABRIC are inherently affected by treatment-selection bias and residual confounding. Consequently, the framework should be viewed as a treatment-support and hypothesis-generating system rather than a clinically deployable recommendation model.

Recent advances in large language models have generated growing interest in oncology-focused clinical decision support, tumor board assistance, and automated medical reasoning. Studies involving GPT-4 and Med-PaLM have reported strong performance in medical examination tasks, clinical reasoning benchmarks, and structured oncology decision-support scenarios (13, 18). However, many of these studies evaluated LLMs using standardized examination-style questions, curated vignettes, or guideline-oriented reasoning tasks rather than individualized treatment-benefit estimation using heterogeneous real-world survival data. This distinction is clinically important because real-world oncology decision-making frequently involves incomplete information, conflicting risk factors, treatment-selection bias, and uncertainty regarding long-term benefit. In contrast to benchmark-style evaluations, the present framework attempted to integrate observational survival modeling with individualized chemotherapy benefit estimation, which represents a substantially more complex and clinically ambiguous task.

Several recent oncology studies summarized in Table 13 reported moderate-to-high agreement between GPT-based systems and multidisciplinary tumor board recommendations (45, 46). For example, Erdat et al. (46) demonstrated strong compatibility between GPT-4 predictions and multidisciplinary tumor board decisions, whereas Alami et al. (45) reported substantially higher agreement in follow-up therapeutic settings than in first-line treatment planning. These findings suggest that LLM performance may improve when the clinical decision space is narrower, more standardized, or more strongly constrained by existing treatment protocols. In contrast, the relatively low agreement observed in the present study (κ=0.13) likely reflects the fundamentally different nature of the task itself. Rather than reproducing guideline-oriented recommendations, the current framework attempted individualized estimation of chemotherapy benefit using uplift modeling and observational survival patterns. This distinction may partially explain why GPT-4 recommendations tended to diverge from uplift-guided treatment allocation, particularly in clinically heterogeneous cases. Another important observation was the tendency of GPT-4 to over-recommend chemotherapy relative to the uplift-based policy. Similar concerns regarding overtreatment tendency, variability, and reduced agreement in clinically complex oncology scenarios have also been reported in prior tumor board simulation studies (47, 48). One possible explanation is that general-purpose LLMs may implicitly prioritize risk-averse or aggressive therapeutic reasoning when uncertainty is high, particularly in cancer-related contexts where undertreatment is clinically undesirable. Additionally, several previous studies used highly structured prompting, guideline-constrained inputs, or institution-specific clinical frameworks, whereas the present study adopted a more flexible few-shot rationale-based prompting design intended to encourage interpretable clinical explanations. Although this approach improved narrative reasoning transparency, it may also have increased variability, prompt sensitivity, and hallucination risk by allowing greater flexibility in GPT-4 response generation.

**Table 13:** Summary of recent studies on large language models for clinical reasoning, oncology decision support, and medical AI prompting strategies.

Study	Clinical task	Model	Prompting strategy	Reported metric(s)	Observation
Singhal et al. (18)	Medical QA and reasoning	Med-PaLM	Instruction prompt tuning	USMLE passing performance reported	Medical alignment and prompting improved clinical reasoning quality.
Nori et al. (13)	Medical challenge problems	GPT-4	Zero-shot prompting	USMLE-level performance reported	GPT-4 demonstrated strong general medical reasoning capability.
Erdat et al. (46)	Multidisciplinary tumor board prediction	GPT-4	Structured clinical prompting	Mean compatibility score = 3.59; Cronbach’s α=0.950	GPT-4 showed high compatibility with MTB decisions, though limitations remained in rare cases.
Karabuğa et al (47)	Tumor board support	ChatGPT-4o	Open-ended prompting	Low agreement reported	Agreement declined in clinically complex oncology scenarios.
Cakir et al. (49)	Prostate cancer treatment recommendation	ChatGPT-4o and DeepSeek-R1	Structured prompting	Cohen’s κ reported	Agreement between AI systems and tumor board decisions varied across tasks.
Alami et al. (45)	Therapeutic decision-making in head and neck oncology	GPT-4	Structured case prompting	κ=0.48 (first-line); κ=0.78 (follow-up)	Agreement was substantially higher in follow-up treatment scenarios.
Schmutz et al. (48)	Molecular tumor board simulation	ChatGPT-4	Structured multidisciplinary prompting	Qualitative evaluation reported	GPT-4 generated clinically relevant therapeutic options but showed variability in evidence prioritization.
Kenaston et al. (50)	Oncology information extraction and reasoning	GPT-4	Zero-shot prompting	F1=0.51	Extraction performance remained limited for complex oncology inference tasks.
Liu et al. (51)	Automated radiotherapy planning	GPT-4Vision	In-context clinical prompting	Clinical plan quality comparison reported	GPT-4-guided planning matched or outperformed several clinical plans.
This Study	Breast cancer chemotherapy decision support	RF + causal uplift + GPT-4	Few-shot rationale prompting	AUC=0.91; κ=0.13	Notable predictive performance but low GPT-policy agreement and chemotherapy over-recommendation tendency.

The influence of prompting methodology on clinical LLM behavior has become increasingly recognized in recent literature. Zero-shot prompting approaches, such as those evaluated by Nori et al. (13), demonstrated strong general medical reasoning capabilities but often relied on highly standardized benchmark tasks. Other oncology-focused investigations incorporated structured prompting, chain-of-thought reasoning, or retrieval-constrained approaches to improve consistency and reduce factual instability. In contrast, the present framework did not employ explicit chain-of-thought enforcement, retrieval-augmented generation, self-consistency prompting, or external guideline retrieval mechanisms. While this simplified prompting strategy allowed flexible rationale generation using clinicopathologic variables, it likely contributed to the lower agreement and greater variability observed relative to more constrained oncology prompting systems. These findings highlight that LLM performance in oncology is not determined solely by model size or architecture, but is also strongly influenced by prompt engineering strategy, clinical context, and task complexity.

The SHAP analysis improved interpretability by identifying ER status, nodal involvement, tumor size, and Nottingham Prognostic Index as the main contributors to the chemotherapy recommendation. These findings were consistent with both established breast cancer treatment principles and the subgroup patterns observed in the uplift analysis, supporting the clinical plausibility of the framework. From a broader clinical perspective, the study demonstrates the potential of combining predictive modeling, causal inference, and explainable AI for more personalized chemotherapy decision-making. Such approaches may help reduce unnecessary treatment exposure while preserving benefit in higher-risk subgroups. In the future, hybrid ML–LLM systems could assist tumor board discussions, clinical prioritization, and decision support, particularly in resource-limited settings where access to specialized expertise remains limited.

Several limitations should be acknowledged when interpreting these findings. The METABRIC dataset is a retrospective cohort collected from an earlier treatment era and may not fully represent current breast cancer management or newer targeted therapies. In addition, because the study relied on observational data, some degree of confounding and treatment-selection bias likely remains despite the use of causal adjustment methods. The study was also affected by class imbalance, simplified handling of missing data, and the use of rule-based extraction for GPT-4 treatment recommendations, which may not fully capture complex clinical reasoning. Another important limitation was the relatively low agreement between GPT-4 and the uplift-based policy, suggesting that LLM recommendations remain sensitive to prompting strategy and may still generate inconsistent or overly aggressive treatment suggestions. Finally, the framework was evaluated using a single retrospective dataset without external or prospective clinical validation. Future work should therefore focus on validating the framework in independent cohorts, integrating multimodal clinical and genomic data, and developing more reliable oncology-specific prompting and clinician-in-the-loop evaluation strategies.

## 6 Conclusion

This study introduced a hybrid framework that combines Random Forest-based prediction, causal survival uplift modeling, and GPT-4 rationale generation to aid chemotherapy decision-making in breast cancer. The Random Forest model achieved balanced accuracy and recall, outperforming several common benchmarks. Uplift modeling measured the variety of benefits and identified subgroups where chemotherapy may be safely deprioritized. GPT-4 provided recommendations based on a rationale that matched clinical factors, improving interpretability even with modest agreement to machine learning predictions. Together, these elements show that merging machine learning with rationale-generating models can offer decision support that is both strong and clear. However, the study relied on a single retrospective cohort, METABRIC. This limits its applicability to populations with different treatment standards and ethnic backgrounds. The study only used clinicopathologic variables. It did not include genomic signatures, imaging biomarkers, or multi-omics, which may affect predictive accuracy. Future work should address these limitations by validating results with independent cohorts, like TCGA and SEER, and using multi-institutional datasets will be essential for establishing applicability. Expanding the feature set to include imaging and molecular profiles may improve the precision of uplift modeling. Methodologically, exploring causal forests, X-learners, and neural survival estimators may better capture diverse treatment effects in cases of censoring.

## Data Availability

The datasets presented in this study can be found in online repositories. The names of the repository/repositories and accession number(s) can be found in the article/Supplementary Material.
